# Is Insulin Receptor Substrate4 (IRS4) a Platform Involved in the Activation of Several Oncogenes?

**DOI:** 10.3390/cancers15184651

**Published:** 2023-09-20

**Authors:** Luis G. Guijarro, Francisco Javier Justo Bermejo, Diego Liviu Boaru, Patricia De Castro-Martinez, Diego De Leon-Oliva, Oscar Fraile-Martínez, Cielo Garcia-Montero, Melchor Alvarez-Mon, María del Val Toledo-Lobo, Miguel A. Ortega

**Affiliations:** 1 Unit of Biochemistry and Molecular Biology, Department of System Biology (CIBEREHD), University of Alcalá, 28801 Alcala de Henares, Spain; 2 Ramón y Cajal Institute of Sanitary Research (IRYCIS), 28034 Madrid, Spain; diego.boaru@edu.uah.es (D.L.B.); diegodleonoliva01@gmail.com (D.D.L.-O.); oscarfra.7@hotmail.com (O.F.-M.); cielo.gmontero@gmail.com (C.G.-M.); mademons@gmail.com (M.A.-M.); miguel.angel.ortega92@gmail.com (M.A.O.); 3 Department of Biomedicine and Biotechnology, University of Alcalá, 28801 Alcala de Henares, Spain; fran16justo@hotmail.es; 4 Department of Medicine and Medical Specialities, Faculty of Medicine and Health Sciences, University of Alcalá, 28801 Alcala de Henares, Spain; patriciadecastro1999@gmail.com; 5 Immune System Diseases-Rheumatology, Oncology Service and Internal Medicine (CIBEREHD), University Hospital Príncipe de Asturias, 28806 Alcala de Henares, Spain; 6 Cancer Registry and Pathology Department, Principe de Asturias University Hospital, 28806 Alcala de Henares, Spain

**Keywords:** IRS4, PI3K/Akt pathway, MAPK pathway, cancer, translational biomarker

## Abstract

**Simple Summary:**

IRS4 (insulin receptor substrate4) belongs to a family of intracellular proteins that include IRS1 and IRS2 and whose physiological function is to transmit the effects of insulin and IGF1 inside the cell. IRS4 is the least studied of this family, and its expression has recently been shown to be increased in many types of cancer. IRS4 serves to connect different signaling pathways, such as MAP kinases and PI3K/AKT. In addition, it has been observed that it is capable of activating several oncogenes, such as BRK (breast tumor kinase) and feline sarcoma-related protein (FER). Increased IRS4 expression in cancer cells may be due to changes in the regulatory region of the gene or increased chromosomal aberrations. Our objective has been to carry out a review to assess the possibility that IRS4 behaves as a meeting point for different oncogene signaling pathways, which we have called the oncogene platform.

**Abstract:**

The IRS (insulin receptor substrate) family of scaffold proteins includes insulin receptor substrate-4 (IRS4), which is expressed only in a few cell lines, including human kidney, brain, liver, and thymus and some cell lines. Its N-terminus carries a phosphotyrosine-binding (PTB) domain and a pleckstrin homology domain (PH), which distinguishes it as a member of this family. In this paper, we collected data about the molecular mechanisms that explain the relevance of IRS4 in the development of cancer and identify IRS4 differences that distinguish it from IRS1 and IRS2. Search engines and different databases, such as PubMed, UniProt, ENSEMBL and SCANSITE 4.0, were used. We used the name of the protein that it encodes “(IRS-4 or IRS4)”, or the combination of these terms with the word “(cancer)” or “(human)”, for searches. Terms related to specific tumor pathologies (“breast”, “ovary”, “colon”, “lung”, “lymphoma”, etc.) were also used. Despite the lack of knowledge on IRS4, it has been reported that some cancers and benign tumors are characterized by high levels of IRS-4 expression. Specifically, the role of IRS-4 in different types of digestive tract neoplasms, gynecological tumors, lung cancers, melanomas, hematological tumors, and other less common types of cancers has been shown. IRS4 differs from IRS1 and IRS2 in that can activate several oncogenes that regulate the PI3K/Akt cascade, such as BRK and FER, which are characterized by tyrosine kinase-like activity without regulation via extracellular ligands. In addition, IRS4 can activate the CRKL oncogene, which is an adapter protein that regulates the MAP kinase cascade. Knowledge of the role played by IRS4 in cancers at the molecular level, specifically as a platform for oncogenes, may enable the identification and validation of new therapeutic targets.

## 1. Introduction

**Figure 1:**
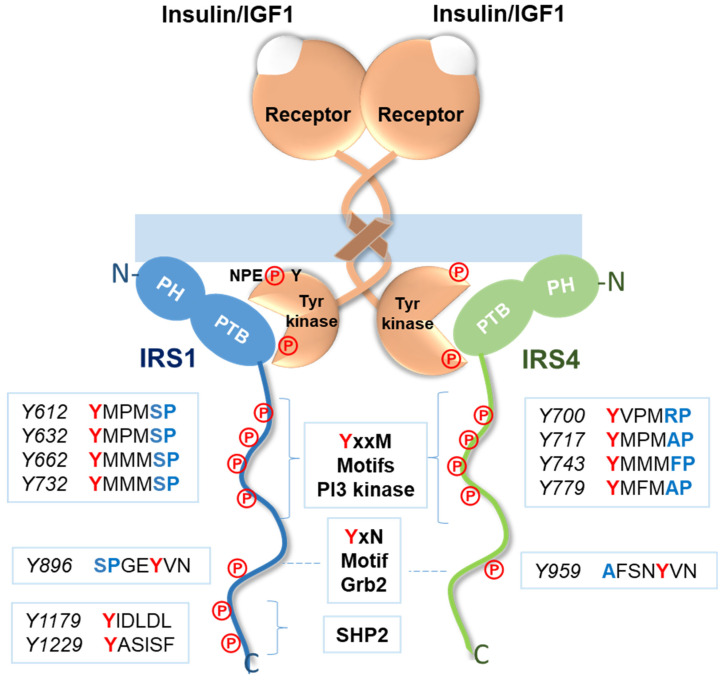
Insulin/IGF1 receptors signaling. Role of IRSs in the insulin and IGF1 signaling pathways. PH = pleckstrin homology domain and PTB = phosphotyrosine-binding domain. ℗ = phosphotyrosine residues.

Insulin receptor substrate-4 (IRS4) is a scaffolding protein that belongs to the IRS family; this group of cytoplasmic proteins integrate and organize multiple cellular processes by transmitting signals from the extracellular to the intracellular domain through transmembrane receptors [1,2]. Additionally, they are the main molecules that control how the body reacts to both insulin and insulin-like growth factor 1 (IGF1) [3]. According to accepted theory, insulin/IGF1 interacts with two highly homologous tetrameric receptors, insulin and IGF1 receptors, with distinct affinities and leading to different biological effects. These receptors have an α-subunit that engages the extracellular ligand and relays the signal to the β-subunit, which shows kinase activity and phosphorylates different tyrosine residues in IRSs [4,5] (Figure 1). There are six members of the IRS protein family, and this diversity raised the possibility that they each play unique physiological roles. Knockout mice have made it possible to establish functional differences among the members of this family. Mice lacking IRS1 exhibited elevated plasma insulin levels and impaired glucose and insulin metabolism as indicated by tolerance tests, suggesting that these mice were mildly insulin resistant [6,7]. On the other hand, mice lacking IRS2 were slightly smaller than normal mice but developed diabetes because of a combination of insulin resistance and impaired proliferation of β-cells in the pancreas [8]. The roles of IRS1 and IRS2 in pathophysiology have been extensively reviewed previously [1,2,9,10,11], so we do not focus on these proteins in this review [11]. IRS3 is a pseudogene that is expressed in mice but not in humans and is therefore not included in this work because we are focusing on human beings [12]. IRS5 and IRS6 belong to this family because their N-terminal regions show a high degree of similarity with those of other members [13,14], but knowledge about these proteins is lacking; therefore, very few bibliographical references are available, and therefore we have not focused on this review. Regarding IRS4, mice that do not express this gene (IRS4-null mice) presented normal fasting and postprandial plasma insulin concentrations and exhibited a slightly altered response to an oral glucose tolerance test, suggesting that IRS4 does not appear to play an important role in carbohydrate metabolism [15]. However, numerous recent articles suggest the importance of IRS4 in carcinogenesis, and to date, no review on this role of IRS4 has been published; therefore, we are addressing it herein.

## 2. Materials and Methods

### 2.1. Compilation of the Bibliography: Search Engines

To collect the necessary information to prepare this review, data were obtained from different bibliographic sources available online. The main search engines used were PubMed, Google Scholar, and Scopus. The most relevant databases used were GeneCards, National Center for Biotechnology Information (NCBI, Bethesda, MD, USA), The Human Protein Atlas (Uppsala and Stockholm, Sweden), BioGrid (Dallas, TX, USA), The Signaling Pathway Project, COSMIC (Catalog of Somatic Mutations in Cancer, Hinxton, Cambridgeshire, UK), UniProt (Lausanne, Switzerland), gnomAD (The Genome Aggregation Database), UCSC (Genomics Institute University of California Santa Cruz), GTEx (Genotype-Tissue Expression Project), ENSEMBL, and SCANSITE 4.0, among others. International databases were used to obtain information on the chromosomal location of IRS4, the structure of the gene, the regulation of its transcription, and the regulation of its translation, and the structural characteristics of the protein, its possible variants, and its posttranslational modifications. Data available online on the IRS-4 interactome were also reviewed.

Another online resource used was the SWISS-MODEL 3D model simulator (https://swissmodel.expasy.org/ (accessed on 5 September 2023)) with which it is possible to obtain an approximate prototype of a protein of interest. This web page also provided data on IRS-4 domains, residues, and proteins with a similar structure.

Key words in parentheses were used for screening. The name of the protein “(IRS-4 or IRS4)” encodes, or the combination of these terms with the word “(cancer)”, was used with selective search engine codes such as “AND” (e.g., “(IRS4) AND (cancer)”). Terms related to specific tumor pathologies (“breast”, “ovary”, “colon”, “lung”, “lymphoma”, etc.) were also used since the possible relationship between IRS4 and oncological diseases is the main objective of this study. Following the same methodology, a search for general information on other members of the IRS family was performed, and information on other proteins, signaling pathways, and data of interest in relation to IRS-4 was collected.

### 2.2. Selection Criteria and Organization of the Results

The selection criteria used were the proven quality of the papers (peer-reviewed publications), its integrity of the main theme of the paper, the focus on understanding the regulatory mechanisms of IRS4 expression or function or the relationship of IRS-4 with pathological processes, especially the relationship with oncological pathology. Other data, especially those related to the structure and regulation of the IRS4 gene, the posttranslational modifications of the protein, and its possible interactions were obtained from database searches. Papers published from August 1997, the date on which the first article on IRS4 appeared in PubMed, to the present (September 2023) were selected. Articles on IRS4 have been searched in Google Scholar and Scopus from 2004 to the present. We excluded articles in which the terms IRS4 or IRS-4 appeared in the context of microorganisms such as *Saccharomyces cerevisiae*, *Rickettsia*, and *Candida albicans*. A total of 137 articles were analyzed.

## 3. Results and Discussion

### 3.1. Signaling Pathways Common to All Members of the IRS Family

**Figure 2:**
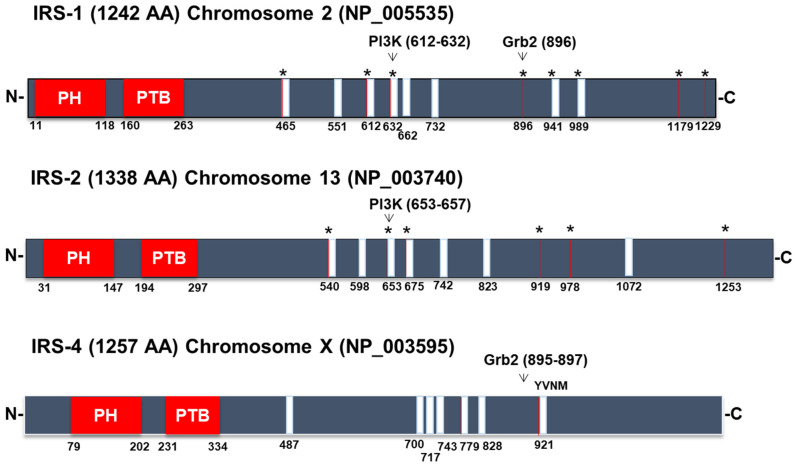
Alignment of the IRS family proteins. PH = pleckstrin homology domain and PTB = phosphotyrosine-binding domain. The asterisk represents tyrosine residues that can be phosphorylated in the presence of insulin. Adapted from [1].

Insulin receptor (IR) and IGF1 receptor (IGF1R) activation involves several signaling pathways that lead to the control of cell metabolism, the cell cycle, and apoptosis [16]. The most extensively studied transducers of these signals in these pathways are IRS1 and IRS2, which are phosphorylated on various tyrosine residues via the tyrosine kinase activity of the β-subunits of both IR and IGF1R [2]. IR and IGF1R carry a sequence in their respective C-terminus composed of asparagine-proline-glutamate-tyrosine (NPEY), and when tyrosine-phosphorylated in the presence of insulin/IGF1, this motif associates with IRS1 to mediate the subsequent intracellular activity triggered by hormones [17] (Figure 1). The IRS1 protein was the first to be identified and cloned [18]. IRS2 was identified as a tyrosine-phosphorylated protein via insulin stimulation in mice after IRS1 was deleted [7]. Despite some domain-to-domain homology, each IRS are structurally unique due to numerous variations. However, every member of the IRS family exhibits one of the most important characteristics that they have in common: a degree of similarity in their N-terminal regions, in which they carry two key domains involved in receptor recruitment, namely, the pleckstrin homology (PH) domain and the phosphotyrosine-binding (PTB) domain [3] (Figure 2). A protein comprising 1257 amino acids is encoded by the IRS4 gene, which is found on the X chromosome [19]. Overall, IRS4 shares only 27% and 29% identical DNA sequences with IRS1 and IRS2, respectively [20]. The IRS4 PTB domain shows 66% and 62% identity with the comparable domain in IRS1 and IRS2 [20] (Figure 2). The PH domain in the N-terminus presents a 49% and 50% homology with IRS1 and IRS2, respectively [20]. The PH domain is necessary for interactions between an adaptor protein and phospholipids in the membrane. IRSs are associated with the cell membrane and come in close proximity to a transmembrane receptor because they primarily bind to membrane phospholipids or the acidic sites in different membrane proteins [21]. Specifically, the aforementioned phosphorylated asparagine-proline-glutamate-tyrosine (NPEpY) sequence is found in IR and IGF1R and is necessary to recognize the PTB domain in IRS and is critical for the hormonal action [17]. While IRS1 and IRS2 are almost ubiquitously expressed, only a small amount of IRS-4 mRNA is expressed in hypothalamus [22] and in skeletal muscle, brain, heart, kidney, and liver [19]. IRS-4 is expressed to a greater extent in the embryonic stages of mice [23].

In less-conserved regions of IRSs, such as in the central domain, short amino acid sequences can recognize the SH2 (Src homology 2) and SH3 (Src homology 3) domains in other adapter molecules and enzymes, which is why they can activate numerous signaling pathways (Figure 2). The p85 regulatory unit of PI3K, Grb2 (growth factor receptor-bound protein 2), SHP2 phosphatase, and phospholipase C are a few examples of specific proteins that carry SH2 domains and bind IRS1 [23,24,25,26] (Figure 2).

These sequences harbor tyrosine residues that can be phosphorylated in the presence of insulin/IGF1 and are called YxxM motifs, which are capable of recruiting and activating PI3 kinase [3] (Figure 1). The C-terminus of IRS1 harbors a YxN motif, which binds to Grb2 and leads to activation of MAP kinases [3] (Figure 1). Finally, IRS1 carries binding sites for the SHP2 enzyme that are not found in IRS4 (Figure 1). The SHP2 protein exhibits tyrosine phosphatase activity, and, as a consequence, its binding to IRS1 causes the dephosphorylation of YxxM motifs, decreasing the activation rate of PI3K [3]. A p85 regulatory unit of PI3K [23,27] and Grb2-binding sites have been found in IRS4 [26]; however, an SHP2-binding site has not been found [23]. Hence, IRS4 can activate the first signaling cascade, but its effect is not diminished by the action of a tyrosine phosphatase, as is the case for IRS1.

Although posttranslational modifications of IRSs do not induce inherent kinase or other enzymatic activity, they assemble signaling molecules that function as adaptor proteins and form enzymatic and nonenzymatic protein docking sites that increase pleiotropic signaling effects within a cell [3]; therefore, IRS proteins integrate a variety of stimuli involving integrins, cytokines, steroids, and hormones in order to regulate the metabolism, growth, survival, and proliferation of cells [28]. The association of p85 with IRS1 leads to the translocation of the enzyme to the membrane and to the formation of phosphorylated phosphoinositides, which in turn leads to the activation of PDK1, PDK2, and, ultimately, AKT [3].

The PI3K/AKT cell signaling system is involved in cytoskeletal remodeling, proliferation, autophagy, apoptosis, and cell survival, among other aspects of cell biology. In human cancers, in which this system is activated via a variety of mechanisms, the PI3K/AKT signaling pathway is one of the most often active signal transduction pathways [29]. Amplification or mutation of PI3K and AKT, activation of growth factor receptors such as the epidermal growth factor receptor (EGFR), loss of function of the tumor suppressor PTEN (phosphatidylinositol-3,4,5-triphosphate-3-phosphatase), and other factors have been frequently implicated in carcinogenesis [30,31].

The AKT family is distinguished by the effects that they mediate to enhance cell signaling. AKT1, AKT2, and AKT3 are three family members that, although they share structural homology, differ from one another in a variety of ways [32]. The AKT family can regulate glycogen, lipid, and protein metabolism via GSK3, AMPK, and mTOR, respectively. However, they are also involved in carcinogenic processes. The AKT1 gene has been linked to processes that contribute to tumor growth. The most significant mTOR (mammalian target of rapamycin) activities in this pathway are regulated by mTORC and GSK3/beta-catenin proteins [33]. mTOR inhibition in cells results in processes, such as autophagy, and a decrease in protein synthesis. AKT functions in the regulation of gene transcription, cell survival, and proliferation through the action of GSK3/beta-catenin. It has been proven that IRS4 causes constitutive hyperactivation of the PI3K/AKT pathway in breast cancer by inducing the phosphorylation and activation of AKT [23].

**Figure 3:**
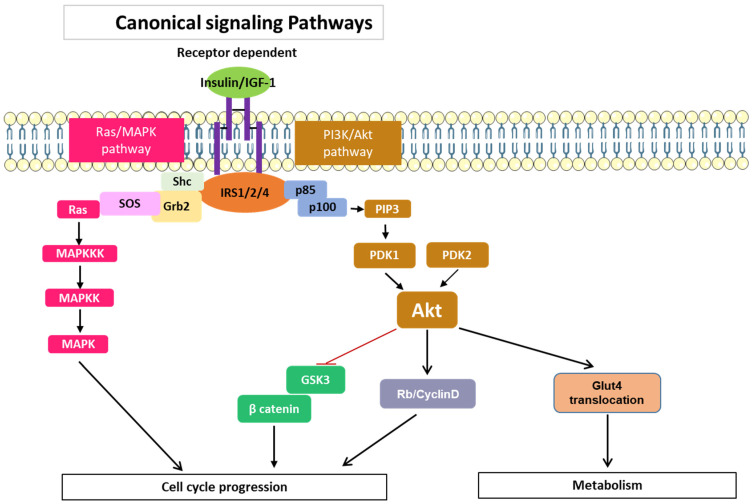
Classical signaling cascades common to IRS1/2/4 involved in cell cycle control and regulation of metabolism. Adapted from [3].

In addition to these aforementioned pathways, IRSs can regulate cell proliferation, differentiation, apoptosis, and stress responses via mitogen-activated kinase-mediated signaling pathway (MAPK) control [34]. Three key kinases, MAPK kinase kinase (MAPKKK), MAPK kinase (MAPKK), and MAPK (ERK), are involved in this pathway (Figure 3). These kinases are phosphorylated and activated in sequential signaling cascade. A transmembrane receptor (such as the IGF-1 receptor) that functions as a tyrosine kinase in response to binding with an external ligand promotes the phosphorylation of several residues in the receptor intracellular domain and in the IRS1 molecule [3]. IRS1 and Grb2 form a complex that recruits the guanine nucleotide exchange factor (GEF) specific for Ras called SOS [3]. As a result, Ras binds GTP and activates the first MAPKKK, named Raf. Downstream Raf phosphorylates MEK (MAPKK) and ERK (MAPK), both of which are thus activated. Among MAPK signal transduction pathways, the Ras/Raf/MEK/ERK pathway is the most significant signaling cascade and is essential for the survival and proliferation of tumor cells [34].

It has been observed in vitro that Grb2 SH2 domains are likely to be recognized by binding motifs in IRS-4, which may stimulate MAPK signaling pathways [35] (Figure 2). Moreover, IRS4 has been shown to be a C-Raf-binding protein in lung cancer [36], enabling MAPK signaling pathway activation. Many cancer cells exhibit irregular proliferation, migration, cell cycle progression, and chemoresistance due to aberrant activation of the PI3K/AKT and Raf/MEK/ERK pathways.

### 3.2. IRS-4-Specific Signaling Pathways

**Figure 4:**
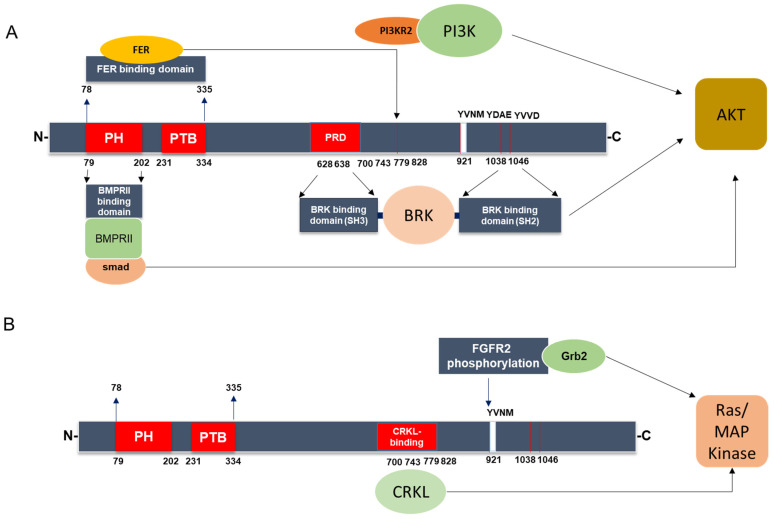
Signaling pathways specific of IRS4. Location of IRS4-specific domains involved in the control of the PI3K/AKT (**A**) and MAP kinase (**B**) cascades. PH = pleckstrin homology domain and PTB = phosphotyrosine-binding domain. PRD = proline rich domain.

IRS4 carries specific structural domains that differentiate it from IRS1 and IRS2. As illustrated in Figure 4, IRS4 can bind to several nonreceptor-regulated intracellular tyrosine kinases, such as FER (feline sarcoma-related kinase) [37], BRK (breast tumor kinase) [38], and other adaptor molecules, such as CRKL [39]. The two former kinases are overexpressed in ovarian cancer [37] and breast cancer [38], respectively, while CRKL is overexpressed in multiple human cancers [40]. CRKL may bind the guanine nucleotide exchange protein SOS, thereby recruiting it to the plasma membrane and activating Ras [41].

Interestingly, the binding of BRK and FER enzymes occurs in nonoverlapping regions. As shown in Figure 4A, FER binds to a large region located at the N-terminus [37], while BRK appears to bind through its SH3 and SH2 domains to a PRD (proline rich domain) and the C-terminus of IRS-4, respectively [38]. At present, the region in IRS4 to which CRKL binds is unknown; however, it is clearly mediated through SH3 and SH2 domains in CRKL [39]. Moreover, IRS4 differs from IRS1/2 in that it can bind to PIK3R2/p85β, one PI3K regulatory subunit considered oncogenic [42]. The mechanism involves FER kinase which phosphorylate IRS4 in Tyr779 creates a binding site to PIK3R2/p85β (Figure 4A). As a key regulatory subunit in the PI3K, recruitment of PIK3R2/p85β to IRS4 is required for activation of the PI3K-AKT signaling pathway and tumorigenesis in ovarian cancer [37].

**Figure 5:**
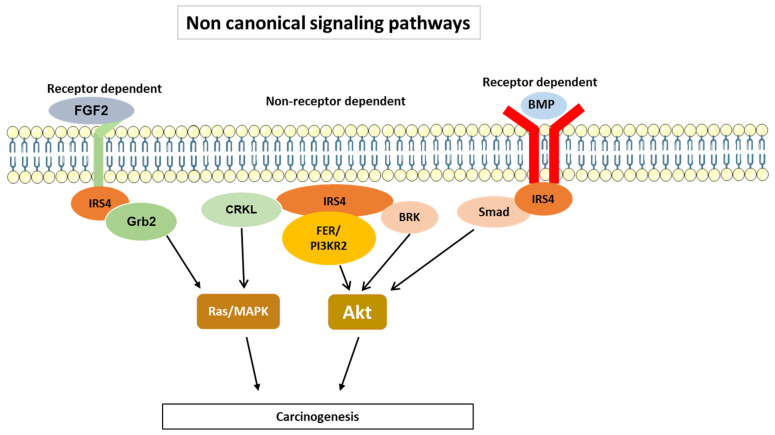
Different signaling pathways in which the IRS4 participates. BMP = bone morphogenetic protein. FGF2 = fibroblast growth factor 2.

In addition, IRS4 is capable of coupling to BMPRII (type II bone morphogenetic protein receptor), which activates AKT [43]. Interestingly, the PH domain in IRS-4 is involved in this association [43]. BRK and FER, as well as the BMPRII pathway, converge to activate AKT (Figure 5), which may be a common event that induces carcinogenesis. Hence, IRS4 may be necessary for tumorigenesis, as it is overexpressed in numerous types of tumors.

These findings indicate that IRS4 is capable of activating the MAPK cascade in a manner similar to that of other members of the IRS family, but it can also activate PI3K through canonical and non-canonical pathways. In the latter pathways, BRK and FER have been implicated in several types of cancer [37,44,45]. Hence, IRS4 overexpression in different cell lines leads to sustained activation of GSK3/βcatenin and Rb/cyclin D [46,47], and Glut4 translocation [48], processes that depend to a greater extent on AKT (Figure 3). Notably, the overexpression of IRS4 in adipocytes caused a higher rate of Glut4 translocation than that mediated by IRS1, and this increased translocation rate did not depend on insulin treatment [48]. The phosphorylation-regulated sites in IRS4 that were predicted via the SCANSITE 4.0 program (selecting the high stringency option) include 19 tyrosine residues; however, not all these predicted residue sites have been confirmed with experiments. In addition to these the sites considered to be common to IRS1/2, two tyrosine sites appear to be specific to IRS4. The aforementioned Y779 is phosphorylated by FER and recruits PI3KR2 (Figure 4A) and Y921, which is phosphorylated through mesenchyme-derived fibroblast growth factor 7 (FGF7), which functions through the FGF type 2 receptor (FGFR2) (Figure 4B) [49,50]. This last system is involved in carcinogenic processes [51].

### 3.3. Regulation of IRS4 Levels

- **At the transcriptional level**

Cis-regulatory elements (CREs) of a gene, or enhancers, can activate gene expression at the transcriptional level, and their effect can be mediated over very long distances, up to one megabase in a primary sequence [52]. For this gene activation to occur, physical contact of enhancers with the promoter of the target gene is necessary. A very high percentage of the genome is made up of CREs, and it is precisely where the most somatic copy-number alterations (SCNAs) accumulate in cancer cells [53]. One of the causes of the transformation of a proto-oncogene into an oncogene is the accumulation of enhancers near the proto-oncogene, which is an event called enhancer hijacking [54]. An analysis of 7416 cancer genomes corresponding to 26 types of tumors that are classified in The Cancer Genome Atlas (TCGA) revealed that IRS4 gene has a large accumulation in CREs in its regulatory region leading to overexpression of IRS4 mRNA and may be involved in numerous types of cancer [55]. Hence, IRS4 has been called a pan-cancer gene [55]. The accumulation of SCNAs in cancer cells affect numerous areas of the genome but particularly to CREs close to IRS4 [55], which has been confirmed by studies of IRS4 expression in lung squamous carcinoma, sarcoma, and cervical squamous carcinoma [55]. At present, we do not know the causes of the enhancer hijacking of IRS4 gene. In mice, it has been suggested that mouse mammary tumor virus (MMTV) inserts part of its genome into the promoter or enhancer sequences of the IRS4 gene, conferring tumorigenic properties [23]. However, this MMTV insertion has not been observed with the other members of the IRS family [23]. It can be hypothesized that, if demonstrated in humans, this mechanism could be leveraged for the prevention of cancer using viral vaccines.

- **Regulation of mRNA stability**

Another way to regulate IRS4 mRNA expression is through miRNAs, which are noncoding RNA molecules that function by binding directly to the 3′ untranslated region (3′-UTR) of a messenger RNA, mediating its degradation [56]. Ectopic expression of miR-493 in malignant tumor melanoma cells led to suppression of cell proliferation and cell cycle arrest by inhibiting the expression of IRS4, among other factors [57]. Furthermore, studies with patients showed that miR-493 levels were reduced in melanoma biopsy samples when compared to normal tissue samples [57].

Another group of noncoding RNAs consists of circular RNAs (circRNAs), which lack a 5′-terminal cap and 3′-terminal poly-A tail and thus form a closed-loop structure [56]. CircRNAs can accumulate in a cell and sponge microRNAs (miRNAs), thereby regulating mRNA stability, or they can interact with RNA-binding proteins and thus regulate gene translation [56]. In gastric cancer, the circRNA hsa_circ_0023409 has been shown to activate the IRS4/PI3K/AKT pathway by sponging miR-542-3p, promoting the development and progression of this type of cancer [58].

- **Regulation of IRS4 protein levels via proteolysis and the involvement of ser/thr phosphorylation**

Using SCANSITE 4.0 (selecting the high stringency option), the presence of several serine/threonine sites in IRS4 have been predicted to be phosphorylated by the enzymes AKT, ERK, GSK3α/β, CK1γ2, CDC2, CDK5; however, only CK1γ2 phosphorylation has been demonstrated with experiments [59].

Casein kinase 1γ2 (CK1γ2) is a member of the serine/threonine kinase (CK1) family that is involved in a variety of functions, including DNA damage repair, cell cycle progression, and cytokinesis [60]. It has recently been shown that CK1γ2 phosphorylates the serine 859 moiety, which promotes the polyubiquitination and degradation of IRS4 through the ubiquitin/lysosome pathway mediated by the carboxyl terminus of the Hsc70-interacting protein (CHIP), ultimately leading to IRS4 degradation in lysosomes in osteosarcoma cells [59]. When CK1γ2 was overexpressed, a decrease in IRS4/AKT levels was observed along with inhibited growth of osteosarcoma cell xenograft tumors in a murine model [59]. It has been confirmed that CK1γ2 functions as a tumor suppressor and participates in a novel regulatory mechanism that promotes the stability of the IRS4 protein at the posttranslational level, as indicated by increased AKT signaling [59]. In the opposite direction, dephosphorylation of IRS4 also promoted its degradation. An example of this is that TNFα promoted the association of PP4 (a serine/threonine phosphatase) with IRS4 and its subsequent dephosphorylation and degradation in a process dependent on a specific inhibitor of PP4 [61]. Unfortunately, the IRS4 domain involved in the latter dephosphorylation mechanism is unknown. Both CK1γ2 and PP4 can be considered enzymatic therapeutic targets for IRS4 regulation in cancer.

### 3.4. Regulation of Cell Growth

IRS4 is an intracellular transductor for hormones, growth factor, and cytokines such as insulin [35], IGF1 [62], GH [63], hepatocyte growth factor [64], leptin [65], and interleukin 4 [66], as has been demonstrated through in vitro experiments. In most of the cited reports, the role of IRS4 was found to be related to the control of cell proliferation mediated by the aforementioned extracellular ligands. In almost all these studies, the increase in proliferation was dependent on PI3K/AKT activation, since it can be inhibited by wortmannin [67]. In the RKO colon cell line, IRS-4 overexpression increased the proliferation rate in a retinoblastoma–cyclin-dependent kinase manner [67], indicating its role in controlling cell cycle checkpoint 1 [67]. In vivo studies on liver regeneration after hepatectomy also suggested an important role for IRS4 in liver regeneration after surgical challenge [68]. The level of functional IRS4 increased during liver regeneration because insulin stimulation in vivo favors the association of IRS4 and the p85 regulatory subunit of PI3K [68]. The function of IRS4 as an anti-apoptotic agent is controversial. In myeloid progenitor cells, IRS-4 mediated AKT signaling after insulin stimulation without promoting an anti-apoptotic effect [69]; however, in pancreatic β-cells (INS-1 cells), IRS4 introduction compensated for the apoptosis induced by a decrease in the IRS2 level [70]. Some studies suggested that IRS4 overexpression in tumor cells makes them more resistant to treatment with trastuzumab and lapatinib [23], as well as actinomycin D [71]. It has recently been shown that IRS4 exerts an effect on tumor cell migration, a very important aspect of tumor growth and metastasis formation. Some circular RNAs accelerated gastric cancer cell growth and metastasis via their regulation of the IRS4/PI3K/AKT pathway [58]. Microscopy studies showed that IRS4 colocalized with SSH1 (Slingshot protein phosphatase-1) in F-actin-rich membrane protrusions [72] of insulin-stimulated cells. SSH1 is a phosphatase that dephosphorylates and reactivates cofilin, a very important pathway in the regulation of the cytoskeleton dynamics and, therefore, in the regulation of cell invasiveness [72].

### 3.5. Overexpression of IRS4 in Cancer Cells

**Figure 6:**
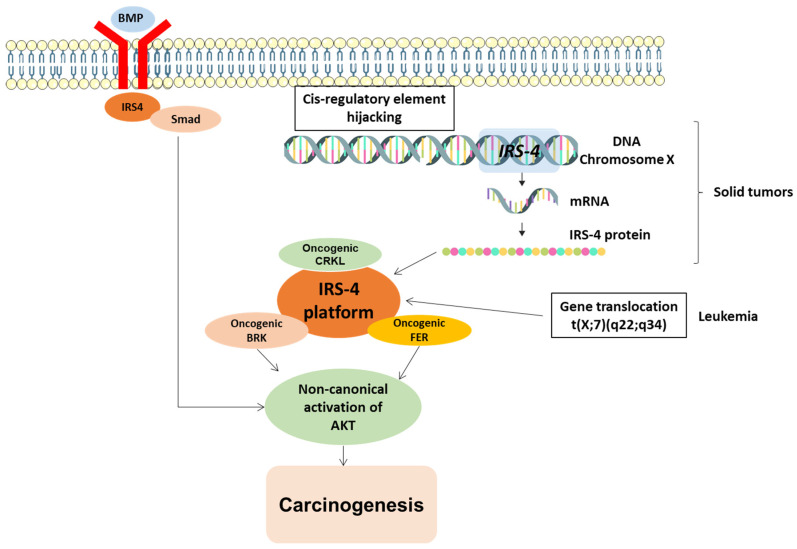
Proposed model of IRS4-mediated oncogenic transformation. General summary on the regulation of IRS4 expression and its role in the control of non-canonical AKT pathways.

Recent research has suggested that IRS4 might function as a proto-oncogene. In large-scale sequencing studies with 7416 genomes from 26 different types of cancer, a number of genes, including IRS4, insulin growth factor 2 (IGF2), SWI/SNF-related matrix-associated actin-dependent regulator of chromatin subfamily A member 1 (SMARCA1), and telomerase reverse transcriptase (TERT), were found to carry accumulated alterations in the cis-elements at their regulatory region; these alterations were largely SCNAs (somatic copy-number alterations), which may favor the expression of these genes [55] (Figure 6).

In an extension of the investigation, IRS4 was again identified as a potential candidate in 1220 malignant tissue samples [73]. Chromatin remodeling, a component of the malignant neoplastic process, shows the potential to influence the gene expression of several genes involved in the behavior of tumor cells [53,54]. In particular, recurrent alterations in cis-elements that enhance tumor cell proliferation have been associated with the upregulation of IRS4 in lung squamous carcinoma [55].

The expression of IRS4 in several human cancers has been evaluated in histological, cytological, and genetic studies, and the findings point to IRS4 involvement in various malignant hematological and solid tumor types. In this section, we explore the role of IRS-4 in different types of tumors to describe its carcinogenic role and related potential translational applications.

### 3.6. IRS-4 in Digestive Tumors

- **Colorectal cancer**

Colorectal cancer (CRC) is the third most prevalent cancer diagnosed in both men and women in the US. Forty-one percent of all CRCs develop in the proximal colon, 22% in the distal colon, and 28% in the rectum [74]. In 2020, it was anticipated that 147,950 people will be diagnosed with colorectal cancer, of whom 53,200 will pass away, with 3640 of these fatalities occurring in patients younger than 50 years old [75]. The other CRC cases, approximately 65% are expected to be sporadic and late-onset colon cancer. Chromosome instability (CIN), alterations in chromosomal number, and structural abnormalities are present in approximately 85% of sporadic CRCs [76,77,78]. A high-frequency microsatellite instability (MSI) is seen in approximately 15% of sporadic colorectal tumors [79,80]. It has been demonstrated that IRS4 is overexpressed in this malignancy, and the nuclear localization of this protein was noted in the study [81]. Moreover, IRS4 was connected to the occurrence of lymph node metastases and tumor invasiveness [81]. A more expansive investigation, however, is required to ascertain the connection between IRS4 overexpression and tumor development and survival. One study revealed that enhanced G1 checkpoint cell cycle proteins were positively correlated with overexpression of IRS4 in cultures of tumor cells of colorectal origin [67]. This overexpression of IRS4 in RKO cells (poorly differentiated colorectal cancer) activated the IGF-1 pathway by forming an IRS4/BRK/pIGF-1R complex, which is in connection with the increase in procaspase 3 levels [47]. Recently, overexpression of procaspase 3 has been reported in a variety of cancer types [82].

Increased procaspase 3 levels in colorectal cancer had been previously associated with phospholipase A2 activation, which in turn triggers prostaglandin E2 and the Wnt/β-catenin pathway [83]. These findings suggest that IRS4 is important to tumor growth mediated through the last pathway. Since CRC modifies the pathways involved cell growth, proliferation, tumor progression, and the emergence of chemotherapy resistance, studying the function of IRS4 in Wnt-mediated pathways will be of significant interest [84,85].

- **Gastric cancer**

Gastric cancer, also known as stomach cancer, originates in the cells that line the stomach and is characterized by unregulated proliferation and division of abnormal stomach wall cells, which can lead to tumorigenesis [86]. In 2020, 27,000 new cases were expected to be diagnosed in the United States [87]. A category of noncoding RNAs known as circular RNAs has been proposed to be crucial to the development of gastric cancer through activation of the PI3K/Akt signaling pathway [88]. In accordance with a recent study, alterations in microRNAs that increase the IRS4 protein may be associated with stomach cancer. In patients with this cancer, hsa_circ_0023409 has been found to be positively correlated with tumor size, histological grade, and tumor grade according to the TNM (tumor, node, and metastasis) classification [58].

This study showed that has_cir_0023409 increased IRS4 levels via sponging miR-542-3p, which controlled IRS4 mRNA levels [58]. Therefore, a decrease in the levels of miR-542-3p implied an increase in IRS4 expression in tumor tissue; in HGC-27 gastric tumor cells, decreasing has_cir_0023409 levels were found to associated with lower cell survival and proliferative potential through activation of the IRS4/PI3K/Akt signaling pathway [58].

- **Hepatocellular carcinoma**

The most prevalent type of primary liver cancer is hepatocellular carcinoma (HCC). These tumors are derived from hepatocytes, the primary class of liver cells critical to optimal liver function maintenance. Malignant liver cells proliferate uncontrollably, which is a feature of hepatocellular carcinoma [89]. More than 80% of initial liver malignancies worldwide are attributed to HCC [90]. There are few studies on IRS4 and liver cancer. Higher levels of IRS4 in the tumor have been linked to elevated cell proliferation and the occurrence of multifocal HCC [46]; this is due to increased levels of the IRS4 transcript [91]. In vitro studies suggest a role of IRS4 in HCC because glypican 3 increases IRS4 phosphorylation and stimulates hepatocellular carcinoma cell proliferation [92]. In addition, IGF-1 has been shown to increase cell proliferation in the HepG2 (a hepatoblastoma cell line) through IRS4 in an ERK-dependent manner, whereas IRS4 knockdown decreased ERK activity and cell proliferation stimulated by IGF-1 [62]. To ascertain the role of this protein in liver cancer, larger studies with patient samples data are needed.

- **Pancreatic cancer**

Pancreatic cancer is one of the most aggressive tumors. According to epidemiological data, its incidence is increasing worldwide and is expected to become the second deadliest type of cancer in the near future [93,94]. The seventh-highest cause of cancer-related death worldwide is attributed to pancreatic cancer, which is classified as the 14th most prevalent malignancy. It is a fatal condition that, by 2030, is expected to rank as the second largest cause of cancer death in the United States [95]. Patients with pancreatic cancer have a poor prognosis in part because 90% of the tumors are discovered late, when they have spread outside of the pancreas and with >50% having developed systematic metastases [96,97].

IRS4 levels are key factors in investigations into pancreatic cancer [98]. Compared to patients who have low/moderate or no IRS4 expression, those who have high IRS4 expression have a 160 times higher mortality risk [99]. Notably, IRS4 causes PI3K/AKT hyperactivation, which has been extensively discussed in terms of its clinical importance in cancer [100]. This pathway affects an extensive variety of biological functions in pancreatic cancer, including metabolic alterations, cell cycle progression and survival, apoptosis prevention, protein synthesis promotion, and genomic instability. PI3K/Akt inhibition has therefore been suggested to be a potential treatment for this particular form of cancer [101]. However, the specific mechanism underlying IRS4 dysregulation in pancreatic cancer is not yet known, and future research may focus on identifying potential reasons underlying IRS4 upregulation.

### 3.7. IRS-4 in Gynecological Neoplasms and Cancers

- **Breast cancer**

Breast cancer is the most prevalent type of cancer in women and the second leading cause of cancer-related death in this group [102,103]. Although there are other varieties of breast cancer, invasive ductal carcinoma, which starts in milk ducts and spreads to surrounding breast tissue, is the most prevalent type. Other less frequently occurring forms include invasive lobular carcinoma, which develops in the lobules that produce milk, as well as ductal carcinoma in situ (DCIS) and inflammatory breast cancer [104]. Recent IARC (International Agency for Research on Cancer) GLOBOCAN 2018 data from 185 countries indicated 2.3 million new cases of breast cancer (11.7%) and a death rate of 6.9% [105]. The incidence of breast cancer is higher in high-income nations (571/100.000) than in low-income countries (95/100.000), which reflects a link between globalization and this disease. Due to the occurrence of multiple biological subtypes reflecting unique molecular profiles and clinicopathological traits, breast cancer is typically referred to as a collection of diseases [106].

Overexpression of IRS4 has been associated with tumor cell proliferation and invasiveness in addition to enhanced insulin/IGF1-independent cell proliferation and constitutive AKT activation [107]. In vitro, mammary epithelial cells overexpressing IRS4 underwent neoplastic transformation. Furthermore, the potential of IRS4 to collaborate with HER2 in the breast cancer-causing process has led to the demonstration of its tumor-causing functions in mice [23]. These studies revealed that increased IRS4 mRNA in breast cancer was associated with triple-negative and HER2-positive tumors, which are associated with shorter survival and increased resistance to anti-HER2 therapy, namely trastuzumab and lapatinib, as determined by analyzing data from mRNA microarrays in previous studies [107].

- **Ovarian cancer**

This cancer describes the growth of malignant cells in the female reproductive organs known as the ovaries, which produce eggs and female hormones. Ovarian cancer is a deadly but relatively uncommon condition that is usually not discovered until it is in a late stage [108]. The most prevalent type of ovarian cancer is epithelial ovarian cancer, which develops in the cells that line the outside of the ovary. There are other types of ovarian cancer. Germ cell tumors, which arise from egg-producing cells, and stromal tumors, which form from connective tissue cells within the ovary, are two additional but less common varieties [109]. The 5-year relative survival rate for all cancers has increased by 20% during the past 30 years [110]. Modern research and improved screening, surgery, and treatment techniques have been primarily credited for the rise in survival rates for many malignancies. Despite these developments, the 5-year survival rate for ovarian cancer remains at only 47%; in contrast, the 5-year survival rate for breast cancer is 85%. Ovarian cancer is the second-most frequent gynecologic cancer-related death in women and the ninth-most frequent cause of cancer death overall [105].

A research investigation revealed that IRS4 is a substrate of the FER tyrosine kinase, which has been implicated in the emergence and spread of ovarian cancer (OVCAR-5) in culture [37]. In xenograft mouse model, the loss of this kinase inhibited the acquisition of metastatic phenotypes because FER-mediated phosphorylation of Tyr779 in IRS4 and enabled the association between IRS4 and PIK3R2/p85β (p85 regulatory subunit of PI3K), and constitutively activated the PI3K/AKT pathway, which supported tumor formation [37]. The inhibition of IRS4 via CRISPR–Cas9 significantly impeded cell growth and almost eliminated AKT activation [37]. On the other hand, IRS4 was markedly overexpressed in ovarian tumors and its upregulation was negatively linked with patient survival and prognosis [37].

- **Leiomyomas**

Leiomyomas are benign uterine tumors made up primarily of smooth muscle cells and a thick extracellular collagen matrix. It has a very minimal likelihood of becoming cancerous, but the cancers can develop when myometrial cells undergo complex chromosomal rearrangements (CRRs), which can result in at least four distinct genetic abnormalities [111].

Chromosome Xq22 abnormalities have been identified in three uterine leiomyomas, and they affect the COL4A5 and COL4A6 genes. In tumors with changes in the COL4A5 and COL4A6 loci, IRS4, which is located next to COL4A5 on the chromosome, was the fifteenth most differentially expressed gene according to an analysis of the differential expression of transcripts [112]. Subsequent research showed that in some leiomyomas, the loss of these genes results in positive IRS4 regulation [113].

### 3.8. IRS-4 in Lung Cancer

Lung cancer is the most prevalent and lethal type of cancer. Although smoking and lung cancer are closely related, nonsmokers can also develop the disease for a number of reasons, such as a genetic predisposition, exposure to second-hand smoke, and environmental contaminants [114]. There are two main types of lung cancer: non-small cell lung cancer (NSCLC), subclassified into lung adenocarcinoma, squamous cell carcinoma, and giant cell carcinoma, accounting for up to 80–85% of all lung cancer cases, and small cell lung cancer (SLCL), representing approximately 10–15% of lung cancer cases. NSCLC and SCLC are usually linked, with NSCLC tumors tending to grow and spread more slowly than SCLC tumors [115]. In a previous study by Weischenfeldt et al. (2017) [55], IRS4 appeared to be overexpressed in NSCLC cells. Subsequent research using the A549 human lung cell line showed that IRS4 accelerated NSCLC tumor growth by activating PI3K/AKT and Ras/MAPK and that it was also associated with resistance to anti-EGFR therapy in this experimental model [36]. Compared to its effect on control cells, a reduction in IRS4 expression prevented up to 70% of A549 lung cancer cell proliferation, migration, and invasion. However, when IRS4 expression was rescued or re-expressed in these cells, the impact on apoptosis was reversed [36].

### 3.9. IRS-4 in Melanoma

Another form of cancer, referred to as melanoma, is composed of melanocytes at the base of the epidermis undergoing a malignant change. It is considered to be one of the most aggressive cancers [116]. There has been a report of IRS4 mutations in melanomas coupled with mutations in a number of PI3K/AKT pathway proteins, and it is believed that these changes may be related to the progression of the illness [117]. Cui et al. (2017) found that the expression of miR-493 was downregulated in the tissues and cells of human melanoma [57]. Typically, miR-493 suppresses tumor growth by preventing cell division and cell cycle progression in human melanoma by inhibition of IRS4 mRNA levels [57]. For this reason, it has been suggested that IRS4 is a promoter of the proliferation, migration, and anti-apoptotic activities of melanocytes, thereby enhancing the progression of melanoma [57].

### 3.10. IRS-4 in Hematological Tumors

T-cell acute lymphoblastic leukemia (T-ALL) is a form of cancer that affects lymphoid cells, particularly T-cell lymphocytes, which play a role in immune system function. During T-ALL, immature T cells in the bone marrow cause excessive production of unusual T cells or lymphoblast [118]. T-ALL was one of the first malignancies in which IRS4 changes were identified. A t(X;7) translocation that causes leukemic neoplasia was discovered through molecular investigations [119]. FISH (fluorescence in situ hybridization) was used to map the breakpoint corresponding in chromosome Xq22.3, which was found be at the beta locus of the T-cell receptor at 7q34. Interestingly, only two genes, COL4A5 and IRS4, which had been close to the breaking point, were reportedly impacted by the regulatory components of TRB (T-cell receptor beta) [119].

The IRS4 gene has been identified as a novel translocation partner to a TCR locus, as indicated via molecular genetic analysis of t(X;7). Regarding the signaling pathways affected, NOTCH1 activated the PI3K pathway in T-ALL cells by downregulating PTEN. These findings suggest that the interaction between NOTCH1 and PTEN may be influenced by IRS4 dysregulation [120].

### 3.11. IRS-4 in Other Malignancies and Proliferative Processes

- **Subungual exostosis**

A subungual exostosis is an uncommon osteocartilaginous tumor that affects the distal phalanx of toes or fingers, and this benign lesion has also been linked to several families with exostoses [121]. Patients with subungual exostosis tend to carry a translocation in chromosomes X and 6-t(X;6), which dysregulates IRS4 transcription. The COL12A1 gene, which is involved in other benign bone–cartilage tumors such as chondromyxoid fibroma, is one of the genes connected to this type of tumor in addition to IRS4. This finding shows that IRS4 may be involved in the formation of the distal skeletal system [122].

- **Meningiomas**

The meninges, protective membranes that encompass the brain and spinal cord, can develop into a meningioma. Meningiomas are typically benign (noncancerous) tumors, but they are occasionally cancerous [123]. The cells that make up the meninges, known as arachnoid cells, show a modest growth rate and, in the beginning, may not exhibit any signs of malignancy. Interestingly, when they grow excessively, these cells can put pressure on nearby brain tissue or cranial nerves, which, depending on where they are located, can cause a variety of symptoms [124].

Although there is no information on the potential involvement of IRS4 in this pathology, sporadic multiple meningioma patients have been shown to carry IRS4 mutations [125]. Further studies are needed to identify any possible consequences from this mutation.

Table 1 presents a summary of all the types of cancer that are described in the paper and the possible role of IRS4 in these pathologies.

## 4. Conclusions

IRS4 is a gene located on the X chromosome that is expressed at high rates during embryonic development but is repressed in most adult tissues. Using in situ hybridization and immunohistochemistry, IRS4 levels have been detected in healthy rat, mouse, and human tissues such as skeletal muscle, brain, heart, kidney, and liver at very low levels.

Moreover, its expression is triggered in several types of cancer and in benign neoplasm (Table 1), indicating an important role for IRS4 in cell proliferation-related processes.

The molecular mechanism of IRS4 action has many similarities to that of IRS1/2 and involves the activation of PI3K/AKT and MAP kinase cascades (Figure 3). However, IRS4 shows different characteristics from those of IRS1/2; for example, IRS4 is able to activate BRK and FER, intracellular tyrosine kinases not regulated by receptors (Figure 4A). In addition, IRS4 associates with the oncogenic adapter protein CRKL (Figure 4B) and the regulatory protein PIK3R2/p85β (Figure 4A and Figure 5), which can activate constitutively MAP kinase and PI3K/AKT pathways, respectively.

Similarly, it has been shown that IRS4 expression is regulated in solid tumors at the transcriptional level by cis-element hijacking in the regulatory regions of the gene or by translocation of IRS4 gene to a very active locus in leukemia (Figure 6). Given the differences between IRS1/2 and IRS4 at the molecular level, knowledge of the signaling pathways controlled by IRS4 may enable the design of new drugs for the treatment of cancer without causing deleterious effects on the regulation of insulin and/or IGF1 signaling cascades.

## Figures and Tables

**Table 1:** Summary of cancer involvement of IRS4.

Type of Tumor	Expression	Mechanism of Dysregulation	Carcinogenic Role	References
Colorectal cancer	Overexpressed	Stimulated G1 checkpoint cell cycle	IRS4/BRK/pIGF-1R complex (Tumor development and survival)	[47,67]
Gastric cancer	Overexpressed	Non-coding microRNAs	has_circ_0023409 (restrict cell survival and proliferative potential)	[58]
Hepatocellular carcinoma	Overexpressed	Not specified	ERK hyperactivation	[46,62]
Pancreatic cancer	Overexpressed	Not specified	PI3K/Akt hyperactivation	[99]
Breast cancer	Overexpressed	Not specified	insulin/IGF1 and AKT activation (tumor cell proliferation and invasiveness)	[107]
Ovarian cancer	Overexpressed	Tyr779 enables IRS4 to attract p85	FER tyrosine kinase	[37]
Leiomyomas	Overexpressed	Mutations in Xq22	CRR (Chromosome rearrangements)	[112,113]
Lung cancer	Overexpressed	Not specified	PI3K/Akt and Ras/MAPK pathways	[36,55]
Melanoma	Overexpressed	Mutations	miR-493 (suppress tumor growth)	[57]
T-cell acute lymphoblastic leukemia	Overexpressed	t(X;7)	CRR (Chromosome rearrangements)	[119]
Subungual exostosis	-	t(X;6)	CRR (Chromosome rearrangements)	[122]
Meningiomas	-	Mutations	-	[125]
Adrenocortical carcinoma	Overexpressed	Induction by growth factors	Endocrine differentiation	[126]
